# Paradoxical Worsening of Tubercular Serpiginous-Like Choroiditis after Initiation of Antitubercular Therapy

**DOI:** 10.4274/tjo.94809

**Published:** 2016-08-15

**Authors:** Ebru Esen, Selçuk Sızmaz, Zeynep Kunt, Nihal Demircan

**Affiliations:** 1 Çukurova University Faculty of Medicine, Department of Ophthalmology, Adana, Turkey

**Keywords:** Tubercular choroiditis, antitubercular therapy, paradoxical worsening, Steroid

## Abstract

In this study, a case with tubercular choroiditis showing severe macular edema and progression of choroidal lesions following initiation of antitubercular treatment is presented and the management of posterior uveitis associated with tuberculosis is evaluated. A 40-year-old female patient was admitted with decreased vision in her right eye and her fundoscopic examination revealed serpiginous choroiditis. It was learned from her medical history that she had taken antitubercular therapy 9 years ago. Mantoux tuberculin skin test showed an area of induration measuring 15 mm and a positive interferon-gamma release assay was documented. Additionally, sequelae lesions due to previous tubercular infection were remarkable on her chest imaging. By excluding other causes of uveitis, the patient was considered presumed ocular tuberculosis and a full standard course of 4-drug antitubercular therapy was initiated. On the seventh day of the treatment existing choroidal lesions showed progression, new foci of choroiditis appeared and severe macular edema occurred. After adding systemic corticosteroid to the treatment, the macular edema resolved and choroidal lesions began to inactivate. In patients with tubercular choroiditis, continued progression may develop after initiation of antitubercular therapy. This paradoxical worsening is thought to be a hyperacute immunologic reaction occurring against antigen load released after antitubercular therapy. This phenomenon may be suppressed by the addition of systemic corticosteroids to the treatment.

## INTRODUCTION

Serpiginous choroiditis is a rare, idiopathic, chronic inflammatory disease. It is characterized by geographic lesions affecting the choroid and the neighboring retinal pigment epithelium (RPE) and outer retinal layers. There are reports in the literature that patients with tuberculosis (TB) infection exhibit similar choroidal signs and it has been emphasized that the two entities must be evaluated separately. The clinical presentation of patients with definite or presumed ocular TB diagnosis has been termed ‘serpiginous-like choroiditis’.^1^ Serpiginous choroiditis is believed to be an autoimmune disease and responds well to systemic steroids; in contrast, treating serpiginous-like choroiditis with steroids can lead to serious systemic and local complications if not accompanied by antitubercular therapy (ATT).

In patients with tuberculous choroiditis, ATT accelerates the healing process and reduces recurrence risk by decreasing the number of bacilli.^2^ In some patients, the rapid destruction of bacilli that occurs with initiation of ATT may cause existing lesions to worsen and new lesions to form. This paradoxical phenomenon occurs more often during the treatment of systemic TB infections, but has also been reported in a few cases of ocular TB. Here we present a case of TB-related choroiditis that exhibited choroidal lesion progression and severe macular edema following initiation of ATT.

## CASE REPORT

**Figure 1:**
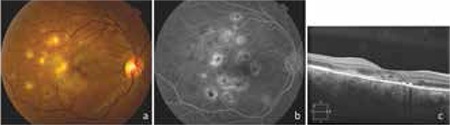
The patient’s clinical findings at presentation: a) Fundus photograph of the right eye showing multiple round, whitish-yellow active subretinal lesions at the posterior pole as well as gray, demarcated sequelae lesions with marginal pigment aggregation, inactive foci and convergent newly activated foci; b) Fundus fluorescein angiography of the right eye showing hypofluorescence in the early arterial phase and hyperfluorescence in the late venous phase due to leakage; c) Optical coherence tomography showing subretinal fluid and hyperreflectivity of the outer retinal layers and choroid

A 40-year-old female patient presented with complaints of vision loss in the right eye beginning 2 months earlier. It was learned that the patient had no known systemic diseases and had received ATT for 6 months 9 years earlier. On ophthalmologic examination, her visual acuity was counting fingers from 2 meters in the right eye and 20/20 in the left eye. Anterior segment examination and intraocular pressure were normal in both eyes. Fundus examination of the right eye revealed multiple round, whitish-yellow, active subretinal lesions and gray sequelae lesions with definite borders and pigment aggregation at the margins. Adjacent to the inactive foci, new foci that tended to converge were noted (Figure 1a). Fundus examination of the left eye was normal. On fundus fluorescein angiography of the right eye, active foci exhibited hypofluorescence in the early arterial phase but were hyperfluorescent in the late venous phase due to leakage. Staining due to pigment epithelium atrophy was observed at the margins of the inactive lesions (Figure 1b). Optical coherence tomography (OCT) revealed hyperreflectivity in the outer retinal layers and choroid as well as intraretinal fluid (Figure 1c).

The patient’s hemoglobin level was 8.3 g/dL and hematocrit was 25.8%; all other biochemical, serologic and rheumatologic test results were normal (antistreptolysin O, C-reactive protein, and rheumatoid factors were within reference ranges; antinuclear antibody, anti-DNA, and human leukocyte antigen B27 tests were negative; no positive results were returned for cytomegalovirus, toxoplasma, herpes simplex virus, hepatitis B or C, human immunodeficiency virus, Salmonella, Brusella, Lyme disease and syphilis tests). Pathergy test was negative. Chest X-ray and computed tomography (CT) showed calcified hilar lymph nodes on the left and peribronchial nodular lesions of soft tissue density in the anterior and posterior segments of the right upper lobe. Mantoux tuberculin skin test (TST) produced an induration of 15 mm at 72 hours, and QuantiFERON TB-Gold test was positive. Following consultation with the Department of Pulmonary Diseases, the patient was considered consistent with previous pulmonary TB with no active pulmonary infection. Systemic investigation by the Department of Infectious Diseases revealed no signs of active or latent extrapulmonary TB. Treatment for iron deficiency was recommended by the Hematology Department. There was no evidence of rheumatologic or dermatologic disease which could cause uveitis.

Due to the presence of choroiditis, TST ≥15 mm, positive QuantiFERON TB-Gold test, previous TB findings on chest X-ray and CT, and with the exclusion of other causes of uveitis, the patient was presumed intraocular TB and diagnosed with serpiginous-like choroiditis. The patient was started on a 4-drug ATT regimen: isoniazid 300 mg/day, rifampicin 600 mg/day, pyrazinamide 30 mg/kg/day, and ethambutol 25 mg/kg/day.

**Figure 2:**
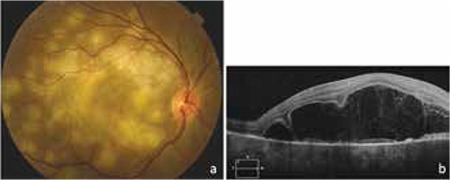
Clinical findings 1 week after initiation of antitubercular therapy: a) Fundus photograph showing progression of existing lesions and multiple new choroiditis foci; b) Optical coherence tomography showing subretinal fluid leading to serous macular detachment

**Figure 3:**
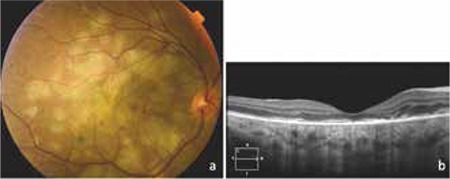
Clinical findings 1 week after adding oral steroid to the treatment regimen: a) Fundus photograph showing choroiditis foci becoming inactive and serous detachment regressing; b) Optical coherence tomography showing minimal intraretinal fluid and disorganized outer retinal layers

On day 7 of treatment the patient’s visual acuity was decreased to counting fingers from 50 cm. Fundus examination revealed progression of the existing lesions, multiple new choroiditis foci, and subretinal fluid leading to serous macular detachment (Figure 2). Oral methylprednisolone (1 mg/kg, 72 mg) was added to the treatment. The subretinal fluid began to regress immediately after the initiation of steroid treatment and had completely resolved 1 week later (Figure 3). After 1 month of treatment, the patient’s visual acuity had increased to 1/10 and a majority of the choroiditis foci were inactive. Extended ATT was planned with 2 months of the 4-drug regimen followed by 10 months of a 2-drug regimen (isoniazid and rifampicin), and reduction of the oral steroid was initiated. After 3 months of treatment, the steroid dose was 24 mg/day. The patient’s visual acuity had increased to 5/10 but new active choroiditis foci were observed. The steroid dose was increased to 54 mg/day and the new foci began to inactivate shortly thereafter. The ATT was continued with the same steroid dose.

**Figure 4:**
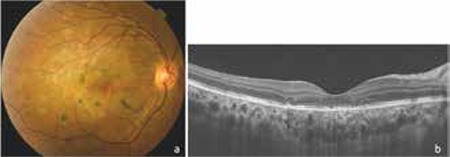
Clinical findings after 6 months of treatment: a) Fundus photograph showing geographic atrophic lesions in the posterior pole and pigment aggregates in the posterior pole; b) Optical coherence tomography showing disorganization of the retinal pigment epithelium and outer retinal layers and inner segment/outer segment band disruption

At the patient’s final follow-up examination after 6 months of treatment, his visual acuity was 10/10. Multiple diffuse pigmented inactive lesions were observed in the fundus. OCT revealed disorganization of the RPE and outer retinal layers and disruption of the inner segment/outer segment band due to photoreceptor layer damage (Figure 4).

## DISCUSSION

A definite ocular TB diagnosis is usually not possible because the direct demonstration of TB bacilli in ocular tissues is difficult. Ocular findings alone are not sufficient to diagnose ocular TB due to the many clinical conditions that can simulate it. When other causes of uveitis have been ruled out, a patient with signs of active or latent TB is referred to as presumed ocular TB.^3^ Especially in endemic areas, patients with findings suggestive of TB uveitis such as granulomatous uveitis, choroiditis, retinal vasculitis, retinal granuloma, and panuveitis should have TB tests included in their systemic evaluation. A TST result of 15 mm or larger is considered positive in vaccinated patients. However, this does not always correspond to a real infection, as the TST can give false positive results, especially in vaccinated individuals. In populations with routine vaccination, the QuantiFERON TB-Gold^®^ (Cellestis Limited, Carnegie, Australia) test may be preferable because it analyzes interferon gamma release and only returns positive results for patients infected with Mycobacterium tuberculosis bacilli.^4^ Like the TST, however, this test cannot discriminate between active and latent infections. Together with medical history and clinical findings, we also confirmed our patient’s infection with TB bacilli using the QuantiFERON test.

Posterior uveitis is the most common clinical manifestation of ocular TB. One of the clinical findings of this manifestation is serpiginous-like choroiditis, which is believed to result from an immune-mediated hypersensitivity reaction against TB bacilli.^2^ This reaction can arise against both bacilli found in the ocular tissue and against remote Mycobacterium tuberculosis antigens.^5^ Treatment response in these patients is relatively slow; it is therefore recommended to start treatment with a 4-drug regimen for 2 months, then continue for 9-12 months with a 2-drug regimen.

When ATT begins destroying TB bacilli, the tubercular antigen load increases and this may further exacerbate the reaction. This paradoxical presentation is the ocular form of Jarisch-Herxheimer reaction that emerges in systemic TB infections like TB meningitis, intracranial tuberculoma, pleural effusion and abdominal TB.^2^ Worsening of clinical findings may be observed in the form of progression of existing lesions and the formation of new lesions. Paradoxical worsening can also occur upon initiation of antibiotic therapy in other ocular infections like syphilis, Lyme’s disease, and leptospirosis. There are other case reports in the literature of ATT inducing clinical exacerbation of ocular TB, similar to our case.^2,6,7,8^ These patients were managed by adding systemic steroids to treatment, increasing the dose of steroid started with ATT, or adding immunosuppressant therapy. Our patient responded well to a high dose of oral steroid and her inflammation rapidly regressed.

There is no standard treatment protocol for TB-related posterior uveitis. Although successful outcomes have been reported with the use of ATT alone, the more common approach is to begin systemic steroid with ATT.^2,9,10,11^ This approach is believed to reduce late stage hypersensitivity-related tissue damage and yield better visual and anatomic improvement. However, it should be noted that steroids can reactivate a latent infection or cause an intraocular infection to spread. Some studies have recommended adding systemic steroids at least 2 weeks after starting ATT.^12^ Treating TB is a long process which requires patience. Especially for the first 2 months, patients must take quite a few pills each day. For patients who have difficulty complying with treatment, the addition of oral steroids may further reduce their compliance with the ATT. For such patients, it may be prudent to make decisions about steroid therapy based on the patient’s clinical course.

Paradoxical worsening may also occur in patients started on both ATT and systemic steroid simultaneously. One reason for this is the very severe inflammation and inadequate suppression. Another reason may be that rifampicin increases steroid metabolism.^13^ In that case, increasing the steroid dose or adding immunosuppressive therapy may resolve the issue. In our patient, new foci of inflammation developed at the steroid maintenance dose of 24 mg/day and the lesions regressed when the dose was increased.

## CONCLUSION

The possibility of the paradoxical phenomenon upon ATT initiation should be kept in mind, particularly for patients with TB-related posterior uveitis, and ATT should not be discontinued with the assumption that the treatment is not effective. Systemic steroid therapy may be beneficial to suppress inflammation and control progression.

### Ethics

Informed Consent: It was taken.

Peer-review: Externally and internally peer-reviewed.

## References

[1] Gupta V, Gupta A, Arora S, Bambery P, Dogra MR, Agarwal A (2003). Presumed tubercular serpiginouslike choroiditis: clinical presentations and management. Ophthalmology.

[2] Gupta V, Bansal R, Gupta A (2011). Continuous progression of tubercular serpiginous-like choroiditis after initiating antituberculosis treatment. Am J Ophthalmol.

[3] Gupta V, Gupta A, Rao NA (2007). Intraocular tuberculosis--an update. Surv Ophthalmol.

[4] Mackensen F, Becker MD, Wiehler U, Max R, Dalpke A, Zimmermann S (2008). QuantiFERON TB-Gold--a new test strengthening long-suspected tuberculous involvement in serpiginous-like choroiditis. Am J Ophthalmol.

[5] Rao NA, Saraswathy S, Smith RE (2006). Tuberculous uveitis: distribution of Mycobacterium tuberculosis in the retinal pigment epithelium. Arch Ophthalmol.

[6] Cheung CM, Chee SP (2009). Jarisch-Herxheimer reaction: paradoxical worsening of tuberculosis chorioretinitis following initiation of antituberculous therapy. Eye (Lond).

[7] Basu S, Das T (2010). Pitfalls in the management of TB-associated uveitis. Eye (Lond).

[8] Neunhöffer H, Gold A, Hoerauf H, Herbort C, Heiligenhaus A, Zimmermann O, Feltgen N (2014). Isolated ocular Jarisch-Herxheimer reaction after initiating tuberculostatic therapy in a child. Int Ophthalmol.

[9] Bansal R, Gupta A, Gupta V, Dogra MR, Sharma A, Bambery P (2012). Tubercular serpiginous-like choroiditis presenting as multifocal serpiginoid choroiditis. Ophthalmology.

[10] Vasconcelos- Santos, Rao PK, Davies JB, Sohn EH, Rao NA (2010). Clinical features of tuberculous serpiginouslike choroiditis in contrast to classic serpiginous choroiditis. Arch Ophthalmol.

[11] Zhang M, Zhang J, Liu Y (2012). Clinical presentations and therapeutic effect of presumed choroidal tuberculosis. Retina.

[12] Mao Y, Peng XY, You QS, Wang H, Zhao M, Jonas JB (2014). Tuberculous uveitis in China. Acta Ophthalmol.

[13] McAllister WA, Thompson PJ, Al-Habet SM, Rogers HJ (1983). Rifampicin reduces effectiveness and bioavailability of prednisolone. Br Med J (Clin Res Ed).

